# Fluconazole plus flucytosine versus fluconazole alone for adults with HIV-associated cryptococcal antigenaemia identified through screening: a multi-centre phase III randomised-controlled trial

**DOI:** 10.1186/s13063-026-09520-x

**Published:** 2026-03-24

**Authors:** Kyla Murphy, Jeremy S. Nel, Mahomed-Yunus Moosa, Douglas P. Wilson, Merika Tsitsi, Sayoki Mfinanga, Sokoine Kivuyo, Denise Kyazze, Nowshad Alam, Graeme Meintjes, Michelle Eriksson, Jack Adams, Colin Menezes, Ebrahim Variava, John Black, Marise Bremer, Vu Quoc Dat, Thuy Le, Charlotte Schutz, Angela Loyse, Rachel M. Wake, David S. Lawrence, Duolao Wang, Shabbar Jaffar, Joseph N. Jarvis, Thomas S. Harrison, Síle F. Molloy, Nelesh P. Govender

**Affiliations:** 1 Wits Mycology Division, School of Pathology, Faculty of Health Sciences, University of the Witwatersrand, Johannesburg, South Africa; 2 Department of Medicine, Wellcome Centre for Infectious Disease Research in Africa, and Neuroscience Institute, University of Cape Town, Cape Town, South Africa; 3 Division of Infectious Diseases, Department of Medicine, Helen Joseph Hospital, University of the Witwatersrand, Johannesburg, South Africa; 4 Division of Infectious Disease, Department of Internal Medicine, Victoria Mxenge Hospital, Nelson R. Mandela School of Medicine. University of KwaZulu-Natal, Durban, South Africa; 5 Department of Internal Medicine, Harry Gwala Regional Hospital, Nelson R. Mandela School of Medicine. University of KwaZulu-Natal, Durban, South Africa; 6 Department of Internal Medicine, Chris Hani Baragwanath Academic Hospital, University of the Witwatersrand, Johannesburg, South Africa; 7 National Institute for Medical Research, Muhimbili Medical Research Centre, Dar es Salaam, Tanzania; 8 Faculty of Medicine and Health Sciences, University of Barcelona, Barcelona, Spain; 9 Infectious Diseases Unit, Department of Internal Medicine, Livingstone Tertiary Hospital, Walter Sisulu University, Gqeberha, South Africa; 10 Department of Internal Medicine, Dora Nginza Hospital, Walter Sisulu University, Gqeberha, South Africa; 11 Department of Medicine and Institute of Infectious Disease and Molecular Medicine, University of Cape Town, Cape Town, South Africa; 12 Blizard Institute, Faculty of Medicine and Dentistry, Queen Mary University of London, London, UK; 13 Infection and Immunity Research Institute, School of Health and Medical Sciences City St George’s, University of London, London, UK; 14 Department of Internal Medicine, Tshepong Hospital, University of the Witwatersrand, Klerksdorp, South Africa; 15 Perinatal HIV Research Unit, Faculty of Health Sciences, University of the Witwatersrand, Johannesburg, South Africa; 16 Department of Medicine, Walter Sisulu University, Gqeberha, South Africa; 17 Department of Medical Microbiology, Groote Schuur Hospital, University of Cape Town, Cape Town, South Africa; 18 Department of Infectious Diseases, Hanoi Medical University, Hanoi, Vietnam; 19 Division of Infectious Diseases and International Health, Duke University School of Medicine, Durham, NC USA; 20 Tropical Medicine Research Center for Talaromycosis in Vietnam, Pham Ngoc Thach University of Medicine, Ho Chi Minh City, Vietnam; 21 Department of Medicine, Institute of Infectious Disease and Molecular Medicine and Wellcome Centre for Infectious Disease Research in Africa, University of Cape Town, Cape Town, South Africa; 22 Department of Clinical Research, Faculty of Infectious and Tropical Diseases, London School of Hygiene and Tropical Medicine, London, UK; 23 Botswana Harvard Health Partnership, Gaborone, Botswana; 24 Department of Clinical Sciences, Liverpool School of Tropical Medicine, Liverpool, UK; 25 Institute for Global Health, University College London, London, UK; 26 MRC Centre of Medical Mycology, University of Exeter, Exeter, UK; 27 Division of Medical Microbiology, University of Cape Town, Cape Town, South Africa

**Keywords:** Human immunodeficiency virus (HIV), Advanced HIV disease (AHD), Cryptococcosis, Cryptococcal antigen (CrAg), Cryptococcal meningitis (CM), Fluconazole, Flucytosine, Randomised controlled trial

## Abstract

**Background:**

Despite the expansion of antiretroviral treatment programmes, the incidence and mortality of HIV-associated cryptococcal meningitis remain high in Africa. Cryptococcal antigen (CrAg) in the blood precedes meningitis. CrAg screening of individuals with advanced HIV disease, combined with pre-emptive fluconazole treatment, reduces the risk of cryptococcal meningitis and associated mortality. However, mortality among individuals with antigenaemia treated with fluconazole is higher than that of CD4-matched individuals with a CrAg-negative test. Autopsy studies have found that cryptococcal disease remains an important cause of death in people with antigenaemia despite pre-emptive antifungal treatment. This suggests a need for more potent treatment for antigenaemia. Flucytosine, combined with fluconazole, is an effective and safe oral treatment for cryptococcal meningitis, and flucytosine has become more widely available as a generic formulation. This trial aims to determine whether combination treatment of fluconazole plus flucytosine is superior to fluconazole monotherapy in reducing all-cause mortality among adults with antigenaemia without evidence of meningitis.

**Methods:**

This multi-centre, open-label phase III randomised-controlled trial embedded in routine CrAg screening programmes in South Africa and Tanzania will compare 14 days of fluconazole (1200 mg/day) plus flucytosine (100 mg/kg/day) to fluconazole (1200 mg/day) alone for the treatment of adults with advanced HIV disease, a blood CrAg-positive test and without evidence of meningitis. Following this 2-week induction therapy, all participants are given fluconazole consolidation and maintenance therapy per local guidelines. The primary endpoint is all-cause mortality at 6 months (superiority analysis). Secondary endpoints include time to all-cause mortality, cryptococcal meningitis-free survival and incidence of symptomatic cryptococcal meningitis, proportion of participants with grade 3 or 4 adverse events, efficacy outcomes by baseline CrAg titre/CrAg semi-quantitative assay score, and health service and household costs per life year saved. A total of 600 participants will be enrolled, 300 per arm, sufficient to detect a 40% relative reduction in mortality with 91% power.

**Discussion:**

An all-oral combination regimen of flucytosine with fluconazole, tested in adults with early cryptococcal disease across a range of baseline CrAg titres, could be an easy-to-administer, safe, and implementable alternative if found to be superior to the current World Health Organization recommended standard of fluconazole monotherapy.

**Trial registration:**

ISRCTN30579828, registered 04 March 2021, and SANCTR DOH-27-122021-6511, registered 06 December 2021.

## Administrative information

**Table:** 

Title {1}	Fluconazole plus flucytosine versus fluconazole alone for adults with HIV-associated cryptococcal antigenaemia identified through screening: a multi-centre phase III randomised-controlled trial Acronym: EFFECT: Efficacy of Flucytosine and Fluconazole as Early Cryptococcal Treatment
Trial registration {2a and 2b}	ISRCTN30579828, registered 04 March 2021 SANCTR DOH-27–122021−6511, registered 06 December 2021
Protocol version {3}	Version 1.5 dated 01 Aug 2024
Funding {4}	The study is funded through the Department of Health and Social Care (DHSC), the Foreign, Commonwealth & Development Office (FCDO), the Medical Research Council (MRC) and Wellcome. This UK-funded award is part of the EDCTP2 programme supported by the European Union
Author details {5a}	Kyla Murphy, Jeremy S. Nel, Mahomed-Yunus Moosa, Douglas P. Wilson, Merika Tsitsi, Sayoki Mfinanga, Denise Kyazze, Nowshad Alam, Graeme Meintjes, Sokoine Kivuyo, Michelle Eriksson, Jack Adams, Colin Menezes, Ebrahim Variava, John Black, Marise Bremer, Vu Quoc Dat, Thuy Le, Charlotte Schutz, Angela Loyse, Rachel M. Wake, David S. Lawrence, Duolao Wang, Shabbar Jaffar, Joseph N. Jarvis, Thomas S. Harrison, Síle F. Molloy* and Nelesh P. Govender* *Equal contribution Kyla Murphy is the corresponding author: kmurphy@witshealth.co.za
Name and contact information for the trial sponsor {5b}	City-St George’s, University of London (CSGUL) Sponsor representative: Subhir Bedi (tel: +44 20 82666397, email: sbedi@sgul.ac.uk)
Role of sponsor {5c}	The sponsor and funders have no involvement except in their capacity as sponsor or funder

## Introduction

### Background and rationale {6a}

#### Burden of HIV-associated cryptococcal disease

Despite the expansion of antiretroviral treatment (ART) programmes in Africa, approximately a third of people living with HIV are not on ART and about one-third seek care after developing advanced HIV disease (AHD) (CD4 count of <200 cells/µl); the incidence of opportunistic infections such as cryptococcal meningitis in this group is high [1]. Furthermore, each year, a substantial proportion of individuals on ART disengage from HIV care, later presenting with AHD and complications such as cryptococcal meningitis [2–4]. Consequently, cryptococcal meningitis rates are not decreasing in most of Africa [5]. *Cryptococcus* is the commonest cause of meningitis among adults living with HIV in Africa and this disease is a key driver of HIV mortality resulting in ~112,000 deaths/year globally with over 63% in Eastern and Southern Africa, and accounting for 19% of all HIV-related deaths in this region [5, 6]. Targeting cryptococcal disease, prior to the development of life-threatening meningitis, can reduce this persistently high HIV-related mortality [7, 8]; this is essential to realising the United Nations agenda to end the HIV epidemic as a global public health threat by 2030 within the framework of the Sustainable Development Goals.

#### Cryptococcal antigen (CrAg) screening and pre-emptive antifungal treatment

Over half of individuals diagnosed with cryptococcal meningitis die within 10 weeks in routine care in Africa [2, 9–12]. However, cryptococcal disease can be detected early, before the development of cryptococcal meningitis, using a simple screening blood test for cryptococcal antigen (CrAg). Untreated, at least half of individuals testing positive [6] for CrAg in blood would progress to develop cryptococcal meningitis or die [7]. Routine CrAg screening, combined with pre-emptive fluconazole treatment, can cost-effectively reduce the risk of cryptococcal meningitis and cryptococcal meningitis-related mortality [7, 13, 14]. CrAg screening has been incorporated into at least 28 national guidelines, and screening programmes are being implemented across Africa.

#### Need for a more potent pre-emptive antifungal treatment

Individuals with antigenaemia who are treated pre-emptively with fluconazole alone have substantially higher mortality compared to blood CrAg-negative individuals with similar CD4 counts (25%–33% mortality versus 9%–15% in different studies [15–17]). In a randomised controlled trial of 1999 participants with AHD (REMSTART trial) [14], conducted in Tanzania and Zambia, there was a threefold excess mortality in screened participants with antigenaemia compared to participants with a blood CrAg-negative test and comparable CD4 cell counts, with a similar elevated risk of death observed from another study [16]. Around a third of those testing blood CrAg-positive may have subclinical cryptococcal meningitis at the time of the screening test [17], yet many individuals decline lumbar puncture and are thus not diagnosed with cryptococcal meningitis. Taken together, this suggests that more effective antifungal treatment is needed to reduce these excess deaths and realize the full benefits of CrAg screening programmes.

#### Combined therapy of fluconazole plus flucytosine

This trial aims to compare the efficacy of an oral combination of fluconazole plus flucytosine with fluconazole alone (the current recommended treatment) in reducing all-cause mortality in adults living with HIV with cryptococcal antigenaemia and no evidence of cryptococcal meningitis in Tanzania and South Africa. The ACTA trial, a phase III, randomised controlled trial, conducted in 5 African countries and including 721 participants with cryptococcal meningitis from 2013 to 2016 [2], showed that fluconazole plus flucytosine for 2 weeks was well-tolerated and as effective as the previous standard treatment regimen of 2 weeks of intravenous amphotericin B deoxycholate plus flucytosine for in-patients with cryptococcal meningitis. Mortality was almost halved compared to historic cohorts treated with fluconazole alone. Although the ACTA trial results cannot be directly generalised to adults with antigenaemia but without meningitis, flucytosine plus fluconazole could be more effective than fluconazole alone in reducing mortality in this group. Importantly, this oral combination could be administered to out-patients without any need for hospitalisation.

#### Impact

A combined oral antifungal treatment for adults living with HIV with a blood CrAg-positive test, without cryptococcal meningitis, identified through screening, has not yet been tested and robust clinical trial data are urgently needed to inform policy and practice. Investigating the efficacy of the addition of flucytosine to the fluconazole monotherapy regimen currently in use could have an important global impact on the reduction of AHD mortality as seen for hospitalised patients with cryptococcal meningitis.

#### Context

The presence of CrAg, a polysaccharide component of the capsule of the fungal organism, which can be detected in blood weeks to months prior to the onset of meningitis, has been shown to be highly predictive of the later development of cryptococcal meningitis and death [7]. This has led to efforts to diagnose and treat early disease (i.e. cryptococcal antigenaemia) to prevent progression to meningitis and death. CrAg screening, using a simple lateral flow assay [18], and treatment of blood CrAg-positive individuals with pre-emptive fluconazole therapy has been shown to be an efficacious, practical and cost-effective approach to reducing mortality [7, 13, 14], with such screening programmes now recommended in at least 28 African countries [8, 19]. Prospective studies have shown that this CrAg screen-and-treat strategy can reduce cryptococcal meningitis rates and contribute to reduced mortality [15]. However, these studies also strongly suggest the full benefits of CrAg screening are not being realised. Current pre-emptive treatment with fluconazole alone may be suboptimal, however, as blood CrAg-positive individuals identified by screening have substantially higher mortality, despite pre-emptive fluconazole treatment, than those individuals with similar CD4 counts screening blood CrAg-negative (25%–32% mortality versus 9%–13% across studies [15–17]). Thus, fluconazole monotherapy, although it saves lives, may not be the optimal treatment for all blood CrAg-positive individuals. Individuals with subclinical cryptococcal meningitis (a third of all who screen blood CrAg-positive) could be identified by lumbar puncture at the time of screening, with hospitalisation for amphotericin B (AmB)-based therapy. However, lumbar punctures are not routinely available in many African settings and even when offered, many individuals decline [15, 16]. Furthermore, lumbar puncture and AmB can only be delivered in well-resourced health facilities. An ongoing trial in Uganda is testing the efficacy of single-dose liposomal amphotericin B (L-AmB) 10 mg/kg plus fluconazole for pre-emptive treatment of individuals with cryptococcal antigenaemia (titres ≥ 160). However, this may be costly and challenging to implement, especially in primary care settings [20].

A study involving minimally invasive autopsies in South Africa indicated that a significant part of the excess mortality is related to inadequately treated cryptococcal disease [17]. All four blood CrAg-positive individuals who died despite fluconazole were found to be cerebrospinal fluid CrAg-positive at the time of death. All had been without signs or symptoms of meningitis, and two, who had agreed to lumbar puncture, were cerebrospinal fluid CrAg-negative at screening [17]. Overall, in the study, cryptococcal disease was still identified as the immediate or contributing cause in 65% of deaths. Furthermore, in the REMSTART trial [15], verbal autopsies were conducted in 12 blood CrAg-positive individuals who died, and eight (67%) deaths were independently attributed to cryptococcal meningitis. Therefore, it is likely that a more potent regimen than fluconazole monotherapy is needed to effectively treat all blood CrAg-positive individuals, including those with subclinical meningitis and those who refuse a lumbar puncture in whom subclinical meningitis cannot be ruled out.

Testing a more efficacious and practical antifungal regimen is thus urgently required. This trial aims to compare the efficacy of an oral combination of fluconazole plus flucytosine with fluconazole alone (the current recommended treatment) in reducing all-cause mortality in adults with AHD and cryptococcal antigenaemia. The ACTA trial demonstrated that combining fluconazole with flucytosine for 2 weeks was safe and non-inferior to 2 weeks of intravenous AmB with flucytosine for patients with symptomatic cryptococcal meningitis [2], with mortality halved compared to historic cohorts treated with fluconazole monotherapy [21]. Subsequently, the AMBITION-cm trial, a phase 3 open-label randomised controlled trial, compared the best-performing ACTA regimen (7 days of AmB plus flucytosine followed by 7 days of high-dose fluconazole) with an experimental regimen consisting of a single high dose of liposomal AmB with 14 days of fluconazole plus flucytosine. Non-inferiority and improved safety and tolerability of the experimental regimen were demonstrated [22] and resulted in updated WHO guidelines recommending the AMBITION-cm regimen as the first-line treatment for HIV-associated cryptococcal meningitis [23].

In South Africa, programmatic data have shown that flucytosine combination therapy was associated with a 53% reduction in-hospital cryptococcal meningitis mortality compared to amphotericin B plus fluconazole in a real-world setting [24].

#### Combination treatment with fluconazole plus flucytosine

Flucytosine was historically expensive and inaccessible across most of Africa, but following the ACTA and AMBITION-cm trial results and inclusion of flucytosine in updated WHO guidelines for cryptococcal meningitis and essential medicines list [25], costs are reducing with the introduction of new generic flucytosine products. Access increased in several African countries through a Unitaid programme, which provided this antifungal medicine to 7 key African countries [26]. Work is also currently underway, through a European Developing Countries Clinical Trials Partnership (EDCTP)-funded project with the Drugs for Neglected Diseases Initiative (DNDi) to develop a sustained-release formulation of flucytosine, which would reduce the need to take medication 4 times daily and aid adherence, particularly in the out-patient setting (ISRCTN18180872, 10.1186/ISRCTN18180872).

Although results to date for the combined oral treatment of fluconazole plus flucytosine for meningitis are promising, they cannot be directly generalised to ambulatory individuals with antigenaemia (without meningitis) who are likely to have a much lower fungal burden in various body compartments. Robust phase III trial data are critical to define the optimal treatment regimens for HIV-associated cryptococcal antigenaemia and inform policy. Should an all-oral combination of flucytosine and fluconazole be found to be superior to fluconazole monotherapy in terms of all-cause mortality reduction, this regimen could be easily administered to out-patients and with few adverse events, as has been observed among in-patients with cryptococcal meningitis.

Despite the risk of cryptococcal disease progression in a proportion of blood CrAg-positive individuals as described above, an early retrospective cohort study in South Africa showed that just over half of blood CrAg-positive individuals (56%) survived without meningitis, with prompt initiation of ART alone [7]. In the REALITY trial, conducted in Uganda, Zimbabwe, Malawi and Kenya, 2013–2015, a package of enhanced prophylaxis including relatively low doses of fluconazole for all those with a CD4 count <100 cells/µL was associated with a reduction in mortality [27].

Among individuals identified with cryptococcal antigenaemia through screening, the risk of having subclinical meningitis and of death is both strongly associated with the antigen titre [28, 29]. Therefore, it may be that the benefit of enhanced antifungal therapy will be more marked in those with higher blood CrAg titres. In addition, a lateral flow semi-quantitative cryptococcal antigen test is now available that could enable implementation of a stratified approach to antifungal therapy based on antigen level.

Therefore, the balance of risks and benefits of more intensive antifungal therapy in the blood CrAg-positive population is not known, and robust data on the impacts of combined treatment, across the range of blood antigen titres, are urgently required.

### Objectives {7}

#### Primary objective

To determine whether combination treatment of fluconazole plus flucytosine for 2 weeks is more effective than standard treatment of fluconazole alone in reducing 6-month all-cause mortality for blood CrAg-positive adults with advanced HIV disease identified through screening.

#### Secondary objectives

To determine:

1. Mortality or meningitis
  - Time to all-cause mortality within first 6 months
  - All-cause mortality at 10 weeks
  - Time to all-cause mortality within the first 10 weeks
  - Cryptococcal meningitis-free survival to 6 months
  - Incidence rate of symptomatic cryptococcal meningitis over 6 months
  - Cryptococcal-related mortality at 6 months
2. Baseline CrAg levels
  - Efficacy outcomes by baseline blood CrAg titre/semi-quantitative assay score
3. Safety and tolerability
  - Tolerability and safety: proportions of participants developing clinical and laboratory-defined grade 3 or 4 adverse events
4. Disability
  - Rates of disability at 6 months
5. Cost-effectiveness
  - Health service and household costs per life year saved

### Trial design {8}

A phase III, multi-centre, open-label randomised-controlled trial embedded in routine screening programmes in South Africa and Tanzania, to compare 2 weeks of combined treatment of fluconazole (1200 mg/day) plus flucytosine (100 mg/kg/day) to fluconazole (1200 mg/day) alone for treatment of blood CrAg-positive individuals, without signs or symptoms of meningitis, with advanced HIV disease.

## Methods: participants, interventions and outcomes

### Study setting {9}

Participants are recruited at 11 sites in South Africa and Tanzania. The site list can be found online at https://www.isrctn.com/ISRCTN30579828.

While the trial sites are mostly district-level/secondary (and some tertiary) hospitals, the majority of participants are recruited from the network of local primary care clinics which refer to each of these facilities. In-patients at these hospital sites may also be enrolled, if eligible.

### Eligibility criteria {10}

#### Selection of study sites and investigators

All study sites are experienced in CrAg screening. The site investigators are qualified by education, training and experience to assume responsibility for the proper conduct of the trial at their site. The investigators are thoroughly familiar with the appropriate use of the investigational product and are certified in, and comply with, the principles of the International Council for Harmonisation guideline for Good Clinical Practice (ICH-GCP) and the applicable regulatory requirements.

#### Participant inclusion criteria

1. Consecutive individuals aged ≥18 years
2. HIV-seropositive
3. CD4 count of <100 cells/µl
4. Blood CrAg test positive within the last 21 days
5. Cerebrospinal fluid CrAg test negative or lumbar puncture not done (declined)
6. Willing to participate in the study

#### Participant exclusion criteria

1. Prior episode of cryptococcal meningitis or cryptococcal antigenaemia
2. Pregnancy (confirmed by urine or serum pregnancy test) or breastfeeding
3. Previous serious reaction to flucytosine or fluconazole
4. Already taking high-dose fluconazole treatment (800–1200 mg/day) for ≥1 week
5. Contraindicated concomitant medications including cisapride and the class of antihistamines including terfenadine
6. HIV-seronegative
7. Clinical symptoms/signs of symptomatic meningitis at any time since CrAg screening, i.e. a progressively severe headache OR a headache and marked nuchal rigidity OR a headache and vomiting OR seizures OR a Glasgow Coma Scale (GCS) score of <15
8. Jaundice
9. Cerebrospinal fluid positive for cryptococcal meningitis (i.e. positive microscopy with India Ink, culture or CrAg test) at any time between the CrAg test and screening for eligibility

#### Late exclusion criteria

1. Division of AIDS (DAIDS) grade 4 abnormalities of platelets, neutrophil count, or creatinine level on baseline bloods:
  - Platelets < 25,000 × 10^6^/L
  - Neutrophils < 0.400 × 10^9^/L
  - Creatinine > 3.5 × upper limit of normal (with standardised upper limit of normal [ULN] of 114 µmol/L, this includes any creatinine level ≥ 400 µmol/L)
2. Microbiological evidence of cryptococcal meningitis on cerebrospinal fluid if full cerebrospinal fluid results are not available at randomisation (e.g. cerebrospinal fluid CrAg test negative but culture on the same sample later returns positive for cryptococcal meningitis)

### Who will take informed consent? {26a}

Site staff who have been trained and delegated by the site principal investigator obtain informed consent, using a standardised participant information sheet and informed consent form (PIS/ICF).

Site staff provide the candidate participant with the PIS/ICF in a language of their choice and offer them time to read the form alone, or with friends or family, including allowing them to take the form home. Once the candidate has made a decision, if they agree to participate, they are asked to sign the form in the presence of the study staff, ensuring that all sections of the form are understood and that any questions the candidate has, have been adequately answered. This process is clearly outlined in a work practice document on which all site staff have been trained and which is available to staff at all times in the Investigator Site File.

Proxy consent is not permitted, i.e. if a candidate is not able to provide informed consent due to illness (Glasgow Coma Scale score of <15) or intellectual impairment, they are excluded. Illiterate individuals can undergo witnessed informed consent, where the witness also signs the PIS/ICF. If a translator is used, they also sign the PIS/ICF.

### Additional consent provisions for collection and use of participant data and biological specimens {26b}

The PIS/ICF specifies the data protection measures to be taken, including the pseudonymisation of all study samples and data by use of a unique participant ID assigned at enrolment/randomisation. Explicit consent is requested for storage of samples, including whether these may undergo genetic testing, and this can be declined without impacting a participation in the trial. Collection and processing of samples for safety evaluations is mandatory and the timing and rationale for these tests is explained in the PIS/ICF.

## Interventions

### Explanation for the choice of comparators {6b}

#### Fluconazole alone 1200 mg daily PO (standard dose ‘control arm’) for 2 weeks

Fluconazole monotherapy for 2 weeks is the currently recommended induction treatment for cryptococcal antigenaemia [23]; the WHO recommends a dose of 800–1200 mg daily. The higher daily dose of 1200 mg is extrapolated from the induction regimen dosing used in the ACTA [2] and AMBITION-cm [22] trials which investigated treatment for cryptococcal meningitis. The REMSTART [15] trial used a lower dose of 800 mg daily for induction treatment of antigenaemia. However, an increase in fluconazole minimum inhibitory concentrations has been observed in cryptococcal isolates from incident cryptococcal meningitis cases in South Africa [30]. Thus, a daily dose of 1200 mg fluconazole was chosen to reduce the risk of breakthrough infection, aligned to the South African national guideline; this dose is also known to be safe and generally well tolerated. 

Fluconazole is administered as 6 × 200 mg tablets or capsules, taken once daily.

### Intervention description {11a}

The intervention (trial) period is the induction phase of treatment for antigenaemia, that is the first 2 weeks of antifungal therapy.

#### Oral fluconazole 1200 mg/day (as above) plus flucytosine 25 mg/kg 6-hourly (intervention arm) for 2 weeks

Flucytosine is currently available in 500 mg capsules or tablets. The dosing schedule is based on a total of 100 mg/kg daily, rounded down to the nearest 500 mg, administered in 4 divided doses.

### Criteria for discontinuing or modifying allocated interventions {11b}

Based on the following baseline laboratory abnormalities, participants may be withdrawn early (late exclusion) from the trial:

1. DAIDS grade 4 neutropenia (absolute neutrophil count <0.4 × 10^9^/L)
2. DAIDS grade 4 renal impairment (absolute creatinine level of ≥400 µmol/L)
3. Microbiological evidence of cryptococcal meningitis, e.g. positive culture or microscopy, where participant was enrolled on a (presumed false) negative cerebrospinal fluid CrAg test

In the above scenarios, all study medication (regardless of treatment arm) is stopped, and the participant is referred to routine care for ongoing treatment.

If a participant is eligible and remains on the trial, study medication may be dose-adjusted or stopped under the following conditions:

1. If a participant withdraws consent to continue study treatment, they are referred to routine care for ongoing treatment of their antigenaemia and HIV.
2. If a participant experiences toxicity thought by the investigator to be related to study medication, the dose may be modified according to clearly defined guidelines in the study work practice documents. Fluconazole doses may be halved, and flucytosine doses may be halved (dosed twice daily) and then reduced further (daily dosing) if needed, with toxicity closely monitored and the dose adjusted up again as soon as possible. For cases in which intolerable toxicity persists despite dose reduction, study medication may be stopped. Alanine transaminase (ALT), renal function and a full blood count with differential are checked at baseline, and full blood count with differential is routinely repeated at day 15. Any abnormalities on day 1 guide additional ad hoc testing at the discretion of the clinician, guided by the work practice documents.
3. As both drugs are renally excreted, baseline renal function guides dosing. If the eGFR (by Cockcroft Gault formula) is <50 ml/min, the fluconazole and flucytosine doses are halved. If the eGFR is <10 ml/min, the flucytosine dose is quartered.

At any time during the study, if a participant develops incident cryptococcal meningitis (not present at baseline), study medication is stopped and the participant is referred to routine care for treatment of their cryptococcal meningitis, though study follow-up continues per protocol.

If a participant becomes pregnant during the intervention period, study medication is stopped immediately and the participant is referred for appropriate antifungal therapy and antenatal care. These participants are followed up beyond the standard 6-month follow-up period to assess pregnancy outcomes.

### Strategies to improve adherence to interventions {11c}

All participants undergo extensive adherence counselling at baseline, including education about cryptococcosis and the importance of adequate antifungal therapy.

Fluconazole is dosed once daily and the timing of this dose is tailored to the participant’s needs, e.g. timing of other medication and work schedule.

Flucytosine is dosed 4 times daily approximately 6 h apart. Participants are asked to complete a medication diary in which they mark off each dose taken.

At days 3 and 9, telephonic follow-up includes adherence counselling and self-reported pill counts.

### Relevant concomitant care permitted or prohibited during the trial {11d}

The trial protocol prohibits the concomitant use of cisapride and the class of antihistamines including terfenadine. Participants receiving these drugs during study screening are excluded. All other medications are allowed, with careful consideration of drug-drug interactions and shared toxicity profiles. Importantly, participants should continue their full package of advanced HIV care, including prophylaxis and treatment for other conditions.

### Provisions for post-trial care {30}

Although the trial intervention is the induction phase (first 14 days of antifungal therapy), the study provides fluconazole consolidation treatment through week 10, to ensure adequate supply to all participants and avoid the impact of potential stock-outs in routine care. In cases in which no routine fluconazole is available, the study may also provide maintenance fluconazole to month 6. Fluconazole maintenance therapy is indicated for varying durations depending on local guidelines (at least 12 months) and is generally widely available at trial sites.

Study participants who develop cryptococcal meningitis during follow-up are treated by routine care; however, if any of the antifungal drugs required are not available, the study provides these. The Trial Management Group is involved in ongoing advocacy for flucytosine access for cryptococcal meningitis in routine care settings.

### Outcomes {12}

#### Primary

All-cause mortality at 6 months after randomisation (proportion, relative risk)—the use of this primary outcome was decided on as a means of minimising bias, given that the trial is open-label.

Justification: If the all-oral combination intervention regimen is found to be superior to the standard of care, this could reduce mortality among individuals who screen positive for cryptococcal antigenaemia without the need for hospital admission. All-cause mortality at 6 months was chosen as the primary outcome for the trial because compared to cryptococcal disease-specific mortality, this endpoint avoids problems such as missing data and changes in disease classification over time. In addition, ascertainment of cause of death among people with advanced HIV disease is difficult based purely on verbal autopsy or record review, with the possibility of multiple co-morbid conditions, some of which may not have been diagnosed ante-mortem.

#### Secondary

1. Time to all-cause mortality within first 6 months, presented in Kaplan-Meier curves per treatment group
2. All-cause mortality at 10 weeks (proportion, relative risk)—sensitivity analysis will also be performed
3. Time to all-cause mortality within the first 10 weeks, presented in Kaplan-Meier curves per treatment group
4. Cryptococcal meningitis-free survival to 6 months
5. Incidence rate of symptomatic cryptococcal meningitis over 6 months—number of events and incident rate by treatment group
6. Cryptococcal-related mortality at 6 months (proportion, relative risk)
7. Tolerability and safety: proportions of participants developing clinical and laboratory-defined DAIDS grade 3 or 4 adverse events
8. Efficacy outcomes (10-week and 6-month mortality) by baseline blood CrAg titre/CrAg semi-quantitative (SQ) assay score
9. Rates of disability at 6 months (using modified Rankin scores)—generalised odds ratio
10. Health service and household costs per life year saved, including annualised health care cost, cost per disability-adjusted life years (DALYs) averted and life years lost, cost offsets and household financial burden per individual.

### Participant timeline {13}



**Table:** 

Event schedule	Screening	Week 1							Week 2								Mth 1	Mth 2.5	Mth 4	Mth 6
Study day	≤D0^~^	D1^~^	D2	D3	D4	D5	D6	D7	D8	D9	D10	D11	D12	D13	D14	D15				
**Consent forms**																				
PIS and signed consent	X	X																		
**Follow-up**																				
Screening	X	X																		
Randomisation		X																		
Clinical review		X														X				
Telephone follow-up				X						X							X	X	X	X
**Clinical labs**																				
HIV testing*^	X																			
CrAg test*^	X																			
CD4 count*^	X																			
Pregnancy test (urine/serum)*^ (1)	X																			
Cerebrospinal fluid testing*^	X																			
Full blood count with differential		X														X				
Serum creatinine		X																		
Serum alanine transaminase		X																		
Urine lipoarabinomannan* (3)	X	(X)																		
**Clinical evaluation**																				
Chest X-ray (2)		X																		
**Research samples—stored at −80 °C**																				
Serum/plasma (5 ml)		X														X				
Whole blood (5 ml)		X																		
Urine (2 ml)		X																		
CSF (2 ml)**	(X)	X																		
**Induction therapy: intervention**																				
Fluconazole plus flucytosine		X	X	X	X	X	X	X	X	X	X	X	X	X	X					
**Induction therapy: control**																				
Fluconazole alone		X	X	X	X	X	X	X	X	X	X	X	X	X	X					

1. For women of childbearing age

2. If clinically indicated (e.g. symptoms of pulmonary tuberculosis). Sputum TB tests will also be performed if clinically indicated, as per national guidelines

3. Urine LAM may be omitted in participants known with TB/on TB treatment

*Part of routine care

^Required for inclusion

^~^Practically, screening and day 1 visit commonly occur on 1 day

**Collected either on D1 or if available, with consent, from routine lumbar puncture CSF sample (also to be collected and stored if participant presents later with symptomatic CM)

### Sample size {14}

Based on evidence from recent studies, we assume that the combined regimen of fluconazole plus flucytosine (intervention arm) could lead to a 40% relative reduction in mortality compared with fluconazole alone. From the ACTA trial [2, 31], the oral combination treatment had a mortality rate of 35% at 10 weeks, compared with 55%–70% mortality of fluconazole alone observed in clinical settings (i.e. a reduction of at least 36% mortality). We assume that 30% of blood CrAg-positive individuals identified during screening and treated with fluconazole alone die. This assumption is inferred from published data, including the REMSTART trial [15] in which 32% of blood CrAg-positive individuals treated with fluconazole (CD4 < 200 cells/µl) died over 6 months of follow-up. In South Africa, mortality was 25% in 2 cohort studies [16, 17].

Using a two-sided *α* = 0.05, a sample size of 540 (270 per arm) will provide 91% power to detect a 40% relative reduction in mortality with fluconazole alone versus flucytosine plus fluconazole (i.e. an observed mortality of 30% vs. 18%, respectively). The trial will also have 81% power to detect a 35% relative reduction if the effect size is smaller, and 83% power if mortality in the fluconazole arm is lower at 25%. Loss to follow-up was 2.5% in the REMSTART trial [15] over 12 months and <1% in the ACTA trial [2] over 10 weeks. A target of 600 participants will be enrolled, to conservatively account for up to 10% loss to follow-up in a pragmatic trial embedded in routine programmes.

### Recruitment {15}

Potentially eligible individuals are identified through local CrAg screening programmes, that is individuals who are HIV-seropositive, have a CD4 count of <100 cells/ml and are blood CrAg-positive. In South Africa, trial staff receive an electronic list of all CrAg-positive results from the national laboratory and from this list, identify potential candidates. In Tanzania, routine staff who receive a CrAg-positive result on their clients refer them to the trial teams at the sites. These individuals are considered by study staff for screening and possible enrolment on the trial and may be contacted and invited to attend a screening visit, during which appropriate screening tests are performed (such as lumbar puncture and urine pregnancy test); these are part of the local CrAg screening programme and not study-specific procedures, but if these have not yet been done by routine care, study staff offer these. Study staff do not routinely repeat blood CrAg testing. During this process, the purpose of these procedures is explained to the individual, in the context of routine care and with reference to the trial. If the individual is found to meet all inclusion criteria and no exclusion criteria, then they are invited to participate in the trial and informed consent is obtained.

## Assignment of interventions: allocation

### Sequence generation {16a}

Individual randomisation takes place using SAS PROC PLAN via a permuted-block randomisation method stratified by site only. Block sizes vary at 4 and 6. The randomisation code has 11-digit numbers: the first 3 digits for protocol (the same across all participants at all sites ‘106’), the next 3 for site, the next 4 for participants within each site, and the final digit is a check-digit to reduce the risk of misidentification between participants.

### Concealment mechanism {16b}

Randomisation lists are created for each site by an independent statistician, and each list is housed on the electronic data capture system (EDC) for that particular site and is inaccessible to trial staff except to randomise the next eligible participant. Randomised allocation for each trial participant is provided to trial staff by extracting the next available randomisation allocation, obtained from the randomisation list for that site housed on the database. Upon confirmation of eligibility criteria and completion and entry of the informed consent details, the EDC system selects the next available randomisation slot and presents the details of the selection to the study doctor/nurse on screen.

### Implementation {16c}

Internally, the EDC selects against an electronic randomisation list prepared in advance by the statistician. The EDC guarantees to make the selection in the natural order of the list, filtering by study site only. Once a selection is made, the randomisation record is tagged with the participant study allocated identifier, date and time of randomisation and other EDC system audit values (username, machine name, etc.). A tagged record cannot be selected more than once. A study doctor makes the final assessment of eligibility for enrolment and participation once all inclusion and no exclusion criteria have been met. Either a study doctor or nurse may then capture this information in the EDC screening form. If the responses to this form indicate eligibility, the informed consent can be recorded and, once completed, the participant is enrolled, and the randomisation arm is assigned automatically and displayed on-screen.

## Assignment of interventions: blinding

### Who will be blinded {17a}

Participants randomised to the control arm (the recommended standard of care in South Africa and Tanzania) receive a simple once-daily dose of fluconazole, while those randomised to the intervention arm will receive flucytosine in addition (4 doses per day). Any potential for bias in this open-label pragmatic trial will be minimised with the use of an objective primary endpoint of all-cause mortality at 6 months. Adding 6-hourly placebos to the control arm would greatly complicate the regimen used in the control arm. This may bias the trial results in favour of the intervention regimen which could be more to do with the complexity of the control arm than the potency/efficacy of the regimens. There is no perfect way of measuring adherence to these antifungal drugs, and so if the control arm is made inferior because of the complexity of the trial design, this would not be known.

The trial statistician is blinded regarding the treatment code when developing the statistical analysis plan and writing the statistical analysis programmes, which are validated and completed using dummy randomisation codes.

### Procedure for unblinding if needed {17b}

The trial is open-label. However, investigators are blinded to aggregate data and unblinding of these aggregate data is only permissible with consent of the Trial Steering Committee.

## Data collection and management

### Plans for assessment and collection of outcomes {18a}

Electronic case report form (eCRF) data collected and validated using the EDC is stored in an electronic database that is protected using a scheme of authentication and encryption. Paper documents, such as clinical notes and administrative documentation, are kept in a secure location (for example locked filing cabinets in a room with restricted access).

The EDC is set up such that a standard visit schedule is pre-populated with all relevant eCRFs per visit (including all required procedures and assessments per protocol), with a visit calendar. When a visit is commenced, all CRFs are immediately available. Additional CRFs are available if needed, and additional unscheduled visits can be created where applicable. Each visit, and each CRF within a visit, can only be completed once per participant.

### Plans to promote participant retention and complete follow-up {18b}

On days 1 and 15, participants are seen in person and compensated for their time, inconvenience and out-of-pocket expenses (mainly transport to site). Beyond day 15, study follow-up is telephonic unless in-person visits are required for clinical indications. The PIS/ICF also informs participants that, should they be unreachable, we may access their medical records to assess vital status and identify any incident cases of cryptococcal meningitis during the 6 months of follow-up. In addition, we may access population (vital status) registers to identify any missed deaths. Compensation for the time taken to complete these telephonic follow-up calls is provided in the form of electronic voucher codes sent to the participant by short message system (SMS).

All compensation for study visits is in line with local compensation models outlined by the regulatory authorities and/or ethics committees.

If no vital status assessment can be made by study close, then the participant is considered lost to follow-up (LTFU).

### Data management {19}

A detailed Data Management Plan has been developed for this trial.

No data are stored on the devices used to access the EDC. Electronic archives are maintained, secured and backed up according to University College London’s (UCL) Archiving Standard Operating Procedure.

The EDC can only be accessed by an authenticated user with user-level permissions. Study staff are provided with mobile internet routers so that live entry is always possible.

The case report forms (CRFs) in the EDC have in-built validations which check within and across forms to minimise errors, and incomplete forms or forms with errors cannot be saved until completed correctly.

Data are downloaded every fortnight and queries run in Stata to identify any missing data, outliers or conflicts, and listings shared with the sites to check and resolve if needed.

### Confidentiality {27}

All participants are assigned a screening identification number (SID) before enrolment and a trial participant identification number (PID), if randomised. All records in the EDC are stored under these SIDs and PIDs. Personal identifiable information values are encrypted and can only be viewed via the EDC and only for validated users. The database is effectively anonymised at rest and in motion. Paper records are anonymised and personal details replaced with the study PID. Only the participant information sheet/informed consent form (PIS/ICF) has both the participant’s name and their study PID. These are kept in a separate file, in a lockable cabinet in an access-controlled study office at each site.

Specimens collected for study storage are collected using the study PID only.

### Plans for collection, laboratory evaluation and storage of biological specimens for genetic or molecular analysis in this trial/future use {33}

Several sub-studies are planned or underway, which make use of stored samples collected during the trial. These include qPCR (cryptococcal nucleic acids) of blood and cerebrospinal fluid, HIV-1 viral load and viral resistance, antiretroviral drug levels, immunogenetics, blood and cerebrospinal fluid factors related to host inflammatory response and organism that might explain the lack of symptoms or signs in subclinical cryptococcal meningitis, and antifungal resistance studies in individuals with antigenaemia who develop symptomatic cryptococcal meningitis while on pre-emptive treatment. Explicit consent is obtained from participants for storage of these samples.

## Statistical methods

### Statistical methods for primary and secondary outcomes {20a}

A detailed, statistical analysis plan (SAP) has been developed. Briefly, the primary analysis will compare the two treatment groups in terms of the proportion of participants who have died from any cause within 6 months from randomisation. A log binomial model (generalised linear model [GLM]) will be used to analyse the primary endpoint, including treatment arm as the sole predictor, to derive the unadjusted relative risk (RR) of 6-month all-cause mortality between intervention and control and the associated 95% confidence intervals (CI).

Covariate adjusted analysis will be performed within the GLM framework including pre-specified covariates: Country, CD4, lumbar puncture at baseline, age, sex, baseline ART status and CrAg titre/semi-quantitative CrAg score group. Subgroup analysis will also be performed for these pre-specified covariates. For the primary outcome, binomial distribution and log link functions will be used, and imputation for baseline missing covariates will be made for the covariate-adjusted analysis. Sensitivity analyses of the primary endpoint making different assumptions for the losses to follow-up and additional per protocol population will be conducted.

Secondary endpoints will also be analysed using GLMs with the treatment arm as the sole predictor. The point estimate of the treatment effect with 95% CI will be derived via specification of a GLM for each secondary outcome, depending on its distribution. For a binary endpoint, the GLM with binomial distribution and log link function will be estimated, and the treatment effects measured as RR. For a continuous outcome, a Gaussian distribution will be assumed, and the identity link function will be used. Correspondingly, the mean difference and its 95% CI between two arms will be calculated. For a count outcome, Poisson distribution and log link function will be used, from which the incidence rate ratio (IRR) and its 95% CI will be computed.

Treatment effects will be analysed and modelled across the range of baseline antigen titres and semi-quantitative titre results as detailed in the Statistical Analysis Plan, and as has been done across the spectrum of meningitis severity in the ACTA and AMBITION-cm trials [32].

Analyses of survival data will be conducted using unadjusted and adjusted Cox regression analysis to calculate the hazard ratio (HR) and 95% CI between the treatment groups. Kaplan-Meier survival curves will also be plotted by treatment group, from which survival rates at 10 weeks and 6 months will be derived. A log-rank test will be performed to compare the survival curves between the treatment groups.

Primary analyses of efficacy outcomes will be based on the intention-to-treat population, and secondary analyses will be based on the per-protocol population. The safety analysis will be on the safety population. The frequency and proportions of participants developing clinical and laboratory-defined side effects (DAIDS grade 3 or 4 adverse events) will be summarised by treatment arm.

### Interim analyses {21b}

No interim analysis is planned. The Data Monitoring Committee reviews safety and outcome data every 6 months and can recommend premature closure or reporting of the trial, or that recruitment to any research arm be discontinued.

### Methods for additional analyses (e.g. subgroup analyses) {20b}

A separate economic analysis plan has been developed. Briefly, an economic analysis will be conducted based on the ITT population. The objective is firstly to determine the relative cost-effectiveness (or efficiency) of the combined therapy versus monotherapy, including screening and early treatment. Standard procedures will be carried out during data collection through the CRF, interviews with staff, during data analysis and probabilistic modelling, using the CHEERS economic guidelines and based on GCP. Secondly, the economic analysis will assess the individual costs of accessing and receiving care, collected through CRFs. The analysis will be performed to demonstrate the societal perspective including both health system costs and household levels on out-of-pocket expenditures. Cost-outcome measures will include annualised health care cost, cost per disability-adjusted life years (DALYs) averted and life years lost, cost offsets and household financial burden per individuals.

### Methods in analysis to handle protocol non-adherence and any statistical methods to handle missing data {20c}

The membership of each analysis set will be determined and documented, and the reasons for exclusion will be given prior to database lock. A summary table will list the individual participants sorted by treatment group and describe their protocol deviation/violation. Three study populations will be considered in the analysis: two to determine efficacy and one for safety.

Intention-to-treat (ITT) population: All participants with valid informed consent will be included according to the treatment to which they are randomised, regardless of whether they prematurely discontinue treatment or are otherwise protocol violators/deviators. Participants that meet late exclusion criteria will not be included in the intention-to-treat population.

Per-protocol (PP) population: a sub-population of the ITT population where participants will be excluded if they were administered incorrect treatment by the study team (i.e. not given the randomised treatment due to study team error), as well as participants who were not adherent to the randomised study regimen, defined as follows: having missed >2 days of either study drug in the first 2 weeks of induction treatment, i.e. >2 doses of fluconazole or >8 doses of flucytosine.

Safety population: This will be defined as all study participants included in the ITT analysis who received at least one day of any of the study interventions.

### Plans to give access to the full protocol, participant-level data and statistical code {31c}

Trial data are suitable for sharing, but the privacy and anonymity of study participants is maintained at all times, with only fully anonymised data made available. The only limits to data sharing are to safeguard research participants’ confidentiality. The proposed procedures for data sharing are set out clearly in the PIS, with current and potential future risks associated with this explained as part of the informed consent process. The TMG ensures that details of the trial are discoverable from the outset through trial registration (ISRCTN), publication of the trial protocol and information on sponsor and partner websites. Upon publication, data that underpin the publications will be made available for access via the CSGUL Research Data Repository (https://sgul.figshare.com/) which has a preservation system to ensure long-term accessibility. It will receive a DOI on publication which will enhance long-term identification and findability of the data. Data will be made available under a creative commons licence. Horizon 2020 Guidelines will be followed, and our commitment to good research data management will be demonstrated by complying with FAIR Guidelines. The access of data will be with the approval of principal investigators and the Trial Steering Committee (TSC). Research data will be made available where possible, with appropriate safeguards where necessary, unless this would breach legislative, regulatory, contractual, ethical or other obligations. Considerations for data sharing will include IPR ownership, ethical, privacy, confidentiality requirements or any legal, regulatory or funding restrictions, as per CSGUL research data management policy. All recipients of the data will be bound by standard data sharing agreements setting out the main responsibilities of external users.

## Oversight and monitoring

### Composition of the coordinating centre and trial steering committee {5d}

The Trial Management Group (TMG) is comprised of the Chief Investigators, supported by senior investigators (clinical and non-clinical) and members of the coordinating centre. The TMG is responsible for the day-to-day running and management of the trial. The TMG meets approximately once weekly online, with one in-person meeting for all investigators at the beginning, middle and end of the trial.

The Trial Steering Committee (TSC) has membership from the TMG (as observers) plus five independent voting members, including the Chair. The role of the TSC is to provide overall supervision for the trial and provide advice through its independent Chair. The ultimate decision for the initiation and continuation of the trial lies with the TSC. Further details of TSC functioning are presented in the TSC charter. All independent TSC members had the opportunity to comment on the protocol as early as possible. The funder has appointed a TSC member with observer status. The lead clinician, South African and Tanzanian national PIs, trial manager and site PIs also attend as observers. This steering committee met at the beginning and then every six months, and is responsible for the overall direction of the research.

### Composition of the data monitoring committee, its role and reporting structure {21a}

An independent Data Monitoring Committee (DMC) has been formed. The DMC is the only group that sees the confidential, accumulating data for the trial. Reports to the DMC are produced by the trial statisticians. The DMC met within 6 months of the trial opening and has met six-monthly since; the frequency of meetings is dictated in the DMC charter. The DMC considers data using the statistical analysis plan and advises the TSC. The DMC can recommend premature closure or reporting of the trial, or that recruitment to any research arm be discontinued. Further details of DMC functioning, and the procedures for analysis and monitoring, are provided in the DMC charter.

### Adverse event reporting and harms {22}

At enrolment, a detailed medical history (including history of exposure and adverse reactions to study medication) and clinical examination are undertaken, and baseline laboratory tests (ALT, creatinine and full blood count with differential) are performed. Any abnormal findings are recorded and graded in line with DAIDS grading conventions. Any worsening from baseline or any new abnormality arising after enrolment until 6 months of follow-up is complete is considered an adverse event. DAIDS grade 1 or 2 adverse events are recorded only in clinical notes but grade 3 or 4 events, as well as all deaths (grade 5), are reported in the EDC system. The severity of the event is determined by the clinical investigator, who also makes an assessment of causality and expectedness as well as seriousness, i.e. whether the event meets criteria for a serious adverse event (SAE) as per ICH-GCP. Non-serious adverse events are reviewed by the relevant site PI and the lead clinician while serious adverse events are reviewed by an additional clinically trained TMG member experienced in the management of this population. The TMG reviewer completes a review form in the EDC indicating their agreement with the site investigator’s assessment. In cases of death, a second TMG member provides additional review and assessment of causation, specifically whether the death is likely due to cryptococcal meningitis.

All adverse events are followed up until resolution or until the participant completes study follow-up, dies or withdraws. Study staff, regardless of whether the event is considered related to study product, facilitate appropriate referral for treatment of the adverse event as necessary.

All adverse events and deaths are reviewed by the DMC and reported to the local regulatory authority and research ethics committee in line with their reporting requirements.

### Frequency and plans for auditing trial conduct {23}

Internal trial monitoring is conducted according to the Trial Monitoring Plan. Site initiation visits facilitated setup at all sites, with monitoring visits early in recruitment, at 50% and at 100% of accrual. Additional ad hoc visits can be triggered by concerns around site performance or participant safety. Real-time monitoring of the EDC data and the electronic Investigator Site Files is also conducted. Details of these monitoring activities are shared with the TMG and sponsor in the form of monitoring reports.

The sponsor has conducted an audit during the course of the trial.

Local regulatory authorities and/or ethics committees may conduct audits, as required.

### Plans for communicating important protocol amendments to relevant parties (e.g. trial participants, ethical committees) {25}

The initial trial protocol was submitted to, and approved by, local regulatory authorities and ethics committees. Any amendments to this protocol are submitted to these bodies for review and approval prior to implementation. Trial registries are updated when the new version has been approved and implemented.

### Dissemination plans {31a}

The ultimate goal of this project is to reduce mortality due to HIV-associated cryptococcal disease through universal access to effective, safe and affordable treatment. We aim to inform global policy and clinical practice through the provision of robust data from a trial which has been designed in partnership with senior policy makers, clinicians and academics in South Africa, Tanzania and the UK, and through discussions with various stakeholders including Ministries of Health (MoH), WHO, Unitaid, MSF, CHAI, DNDi and the pharmaceutical industry. The Trial Management Group (TMG) meets with MoH colleagues regularly to understand how best to disseminate findings for policy considerations and the steps needed for guideline changes if the results are in favour of a combined regimen. On the other hand, if findings from this trial do not support a combination regimen, this will still constitute an important advancement of knowledge in the field and necessarily refocus future research on effectively implementing current and alternative care pathways and treatment options for reducing mortality amongst those living with advanced HIV disease.

Dissemination of results: Results will be presented at high-impact regional and international conferences, published in a high-impact medical journal and disseminated through press releases, CSGUL’s and partners’ websites (a specific trial website has also been created, https://www.witsmycology.co.za/projects/EFFECT). Social media platforms (https://x.com/effecttrial, @effecttrial.bsky.social‬) and trial newsletters will also be used. Results will be rapidly disseminated to regional and international treatment guideline committees, leveraging the fact that trial investigators are members of the following guideline writing committees: Botswana and SA government, SA HIV Clinicians Society, WHO, Infectious Diseases Society of America and the European Confederation for Medical Mycology/International Society for Human and Animal Health (ECMM/ISHAM). In Tanzania, a policy briefing workshop is planned for the end of the trial. The site PI will invite members of MoH, local government, implementing partners and global funders to discuss policy impact. In South Africa, the national and provincial government will be briefed about trial findings and policy impact through existing structures, including the Advanced HIV Disease Task Team.

In addition, in Tanzania and at each of the South African sites, a dissemination meeting with additional attendees including clinicians from local facilities, district and regional medical officers, and health management teams will be informed about the findings.

Community engagement: Members of civil society and patient advocates participate in the Trial Steering Committee (TSC), providing input in particular on local capacity building and continued advocacy efforts around flucytosine and the TMG plans to maintain close collaboration with patient and community representatives to inform approaches of scale-up that are both feasible and acceptable to patients and communities. South Africa's Treatment Action Campaign (TAC) strongly supports the need for this evidence and has delegated a senior representative to the TSC. In addition, TAC is involved in advocacy efforts for flucytosine, minimising stock-outs at the facility level, and improving patient and community literacy for cryptococcal disease.

Local staff will benefit from working with trained and specialised teams and will be key, as leading sites, in any scale-up efforts.

## Discussion

As far as possible, this trial is conducted in a pragmatic way, embedded in routine screening programmes. Through the trial team’s intimate understanding of the routine care CrAg screening programmes, and optimisation of these, the impact of this trial is likely to extend to improvements in the routine care of individuals with early cryptococcal disease. The trial provides consolidation fluconazole therapy through week 10 to minimise the impact of stock-outs and supports routine care in the management of trial participants who develop cryptococcal meningitis while on trial. Investigating the effectiveness and safety of the addition of flucytosine to the fluconazole treatment currently in use could have an important global impact on the reduction of AHD mortality, as has been seen for in-patients with meningitis.

## Trial status

The trial opened to recruitment in South Africa in October 2022, and November 2022 in Tanzania. Recruitment is expected to be completed in October 2025.

## Data Availability

The final trial dataset will be available to the TMG and the trial statistician for initial analysis. The results will be presented to the DMC and TSC, and data made available to them on request. Journal articles will be written and submitted at the end of the project. At this point, data that underpin these papers will be made available for access as detailed above. We will protect the privacy and anonymity of the trial participants (only the fully anonymised database will be available). The study team will have exclusive use of the data until the trial results are published in a peer-reviewed journal (anticipated to be within a year of trial completion).

## Abbreviations

AHD: Advanced HIV disease
ALT: Alanine transaminase
AmB: Amphotericin B deoxycholate
ART: Antiretroviral therapy
CHAI: Clinton Health Access Initiative
CI: Confidence interval
CM: Cryptococcal meningitis
CrAg: Cryptococcal antigen
DAIDS: Division of Allergy and Infectious Diseases
DNDi: Drugs for Neglected Diseases Initiative
EDC: Electronic data capture system
EFFECT: Efficacy of Flucytosine and Fluconazole as Early Cryptococcal Treatment
FBC: Full blood count
GCS: Glasgow Coma Scale
GLM: Generalised linear model
HIV: Human immunodeficiency virus
HR: Hazard ratio
IRR: Incident rate ratio
LAM: Lipoarabinomannan
LP: Lumbar puncture
MSF: Médecins Sans Frontières (Doctors Without Borders)
MoH: Ministry of Health, Tanzania
PCR: Polymerase chain reaction
PJP: *Pneumocystis jirovecii* Pneumonia
qPCR: Quantitative PCR
RR: Relative risk
SQ: Semi-quantitative
SSA: Sub-Saharan Africa
TB: Tuberculosis
WHO: World Health Organisation
